# Supplementary material for: Allosteric regulation and crystallographic fragment screening of SARS-CoV-2 NSP15 endoribonuclease
Source: Nucleic Acids Res. 2023 Apr 28;51(10):5255–70. doi: 10.1093/nar/gkad314 (PMC10250223; doi:10.1093/nar/gkad314)
Supplement: gkad314_Supplemental_Files [file gkad314_supplemental_files.zip › Supplementary datasets.zip!/231/Refine_8/01-BUSTER/Cycle-1/shell.01/R_Rfree.html]

# Rxpct factors in resolution bins at cycle 0


|  |  |  |  |  |  |  |  |  |
| --- | --- | --- | --- | --- | --- | --- | --- | --- |
|  | **<d> (Å)**  Rxpct\_Work | Rxpct\_Free | Rxpct\_All | |  |  |  |  |
|  | 13.1576 | 0.3255 | 0.3705 | 0.3274 |  |
|  | 7.5965 | 0.2966 | 0.2982 | 0.2967 |  |
|  | 5.8843 | 0.3022 | 0.2773 | 0.3012 |  |
|  | 4.9731 | 0.2014 | 0.1979 | 0.2012 |  |
|  | 4.3859 | 0.1691 | 0.1506 | 0.1681 |  |
|  | 3.9672 | 0.1850 | 0.1731 | 0.1844 |  |
|  | 3.6493 | 0.2097 | 0.2121 | 0.2099 |  |
|  | 3.3973 | 0.2235 | 0.2128 | 0.2229 |  |
|  | 3.1912 | 0.2075 | 0.2040 | 0.2073 |  |
|  | 3.0186 | 0.1988 | 0.2124 | 0.1994 |  |
|  | 2.8712 | 0.2089 | 0.1826 | 0.2076 |  |
|  | 2.7435 | 0.1964 | 0.1928 | 0.1962 |  |
|  | 2.6315 | 0.1938 | 0.2100 | 0.1948 |  |
|  | 2.5322 | 0.2012 | 0.1856 | 0.2003 |  |
|  | 2.4433 | 0.2028 | 0.1920 | 0.2023 |  |
|  | 2.3632 | 0.2116 | 0.2074 | 0.2114 |  |
|  | 2.2904 | 0.2091 | 0.1940 | 0.2084 |  |
|  | 2.2240 | 0.2142 | 0.2157 | 0.2143 |  |
|  | 2.1631 | 0.2167 | 0.2321 | 0.2174 |  |
|  | 2.1069 | 0.2644 | 0.2572 | 0.2639 |  |
|  | **Overall** | 0.2191 | 0.2130 | 0.2188 |  |

# Rxpct factors in resolution bins at cycle 1


|  |  |  |  |  |  |  |  |  |
| --- | --- | --- | --- | --- | --- | --- | --- | --- |
|  | **<d> (Å)**  Rxpct\_Work | Rxpct\_Free | Rxpct\_All | |  |  |  |  |
|  | 13.1576 | 0.3242 | 0.3674 | 0.3260 |  |
|  | 7.5965 | 0.2949 | 0.2979 | 0.2951 |  |
|  | 5.8843 | 0.2989 | 0.2738 | 0.2979 |  |
|  | 4.9731 | 0.2002 | 0.1982 | 0.2001 |  |
|  | 4.3859 | 0.1683 | 0.1515 | 0.1674 |  |
|  | 3.9672 | 0.1844 | 0.1741 | 0.1839 |  |
|  | 3.6493 | 0.2100 | 0.2128 | 0.2102 |  |
|  | 3.3973 | 0.2217 | 0.2104 | 0.2211 |  |
|  | 3.1912 | 0.2056 | 0.2045 | 0.2056 |  |
|  | 3.0186 | 0.1975 | 0.2088 | 0.1980 |  |
|  | 2.8712 | 0.2071 | 0.1848 | 0.2060 |  |
|  | 2.7435 | 0.1962 | 0.1960 | 0.1962 |  |
|  | 2.6315 | 0.1937 | 0.2129 | 0.1948 |  |
|  | 2.5322 | 0.1999 | 0.1875 | 0.1991 |  |
|  | 2.4433 | 0.2034 | 0.1888 | 0.2027 |  |
|  | 2.3632 | 0.2127 | 0.2131 | 0.2127 |  |
|  | 2.2904 | 0.2104 | 0.1956 | 0.2097 |  |
|  | 2.2240 | 0.2163 | 0.2208 | 0.2165 |  |
|  | 2.1631 | 0.2191 | 0.2365 | 0.2199 |  |
|  | 2.1069 | 0.2665 | 0.2550 | 0.2658 |  |
|  | **Overall** | 0.2182 | 0.2133 | 0.2180 |  |

# Rxpct factors in resolution bins at cycle 2


|  |  |  |  |  |  |  |  |  |
| --- | --- | --- | --- | --- | --- | --- | --- | --- |
|  | **<d> (Å)**  Rxpct\_Work | Rxpct\_Free | Rxpct\_All | |  |  |  |  |
|  | 13.1576 | 0.3387 | 0.3870 | 0.3408 |  |
|  | 7.5965 | 0.3146 | 0.3146 | 0.3146 |  |
|  | 5.8843 | 0.3126 | 0.2877 | 0.3116 |  |
|  | 4.9731 | 0.2064 | 0.2093 | 0.2065 |  |
|  | 4.3859 | 0.1689 | 0.1549 | 0.1681 |  |
|  | 3.9672 | 0.1843 | 0.1751 | 0.1839 |  |
|  | 3.6493 | 0.2090 | 0.2137 | 0.2092 |  |
|  | 3.3973 | 0.2230 | 0.2114 | 0.2224 |  |
|  | 3.1912 | 0.2071 | 0.2110 | 0.2073 |  |
|  | 3.0186 | 0.1998 | 0.2093 | 0.2002 |  |
|  | 2.8712 | 0.2061 | 0.1818 | 0.2049 |  |
|  | 2.7435 | 0.1961 | 0.1974 | 0.1962 |  |
|  | 2.6315 | 0.1959 | 0.2093 | 0.1967 |  |
|  | 2.5322 | 0.2008 | 0.1916 | 0.2002 |  |
|  | 2.4433 | 0.2022 | 0.1869 | 0.2015 |  |
|  | 2.3632 | 0.2076 | 0.2130 | 0.2078 |  |
|  | 2.2904 | 0.2068 | 0.1916 | 0.2061 |  |
|  | 2.2240 | 0.2125 | 0.2117 | 0.2125 |  |
|  | 2.1631 | 0.2154 | 0.2335 | 0.2163 |  |
|  | 2.1069 | 0.2563 | 0.2537 | 0.2562 |  |
|  | **Overall** | 0.2214 | 0.2177 | 0.2212 |  |

# Rxpct factors in resolution bins at cycle 3


|  |  |  |  |  |  |  |  |  |
| --- | --- | --- | --- | --- | --- | --- | --- | --- |
|  | **<d> (Å)**  Rxpct\_Work | Rxpct\_Free | Rxpct\_All | |  |  |  |  |
|  | 13.1576 | 0.3172 | 0.3465 | 0.3184 |  |
|  | 7.5965 | 0.2790 | 0.2987 | 0.2802 |  |
|  | 5.8843 | 0.2751 | 0.2717 | 0.2750 |  |
|  | 4.9731 | 0.1920 | 0.2087 | 0.1930 |  |
|  | 4.3859 | 0.1647 | 0.1521 | 0.1640 |  |
|  | 3.9672 | 0.1819 | 0.1880 | 0.1822 |  |
|  | 3.6493 | 0.2054 | 0.2294 | 0.2066 |  |
|  | 3.3973 | 0.2138 | 0.2224 | 0.2142 |  |
|  | 3.1912 | 0.2014 | 0.2112 | 0.2019 |  |
|  | 3.0186 | 0.1953 | 0.2041 | 0.1957 |  |
|  | 2.8712 | 0.1999 | 0.1939 | 0.1996 |  |
|  | 2.7435 | 0.1894 | 0.1947 | 0.1897 |  |
|  | 2.6315 | 0.1875 | 0.2130 | 0.1890 |  |
|  | 2.5322 | 0.1912 | 0.1757 | 0.1903 |  |
|  | 2.4433 | 0.1943 | 0.1890 | 0.1940 |  |
|  | 2.3632 | 0.2001 | 0.2075 | 0.2004 |  |
|  | 2.2904 | 0.1996 | 0.1928 | 0.1992 |  |
|  | 2.2240 | 0.2079 | 0.2170 | 0.2083 |  |
|  | 2.1631 | 0.2106 | 0.2297 | 0.2115 |  |
|  | 2.1069 | 0.2546 | 0.2454 | 0.2540 |  |
|  | **Overall** | 0.2105 | 0.2167 | 0.2108 |  |
